# The effect of bi-annual community-directed treatment with ivermectin on the incidence of epilepsy in onchocerciasis endemic villages in South Sudan: a study protocol

**DOI:** 10.1186/s40249-018-0496-2

**Published:** 2018-11-14

**Authors:** Gasim Abd-Elfarag, Makoy Yibi Logora, Jane Y. Carter, Morrish Ojok, Jackson Songok, Sonia Menon, Ferdinand Wit, Richard Lako, Robert Colebunders

**Affiliations:** 1 Department of Global Health, Academic Medical Center, University of Amsterdam, Amsterdam, The Netherlands; 2 Amsterdam Institute for Global Health and Development, Amsterdam, The Netherlands; 3 Neglected Tropical Diseases Unit, Ministry of Health, Juba, Republic of South Sudan; 4 Amref International University, Nairobi, Kenya; 5 Amref Health Africa, Juba, South Sudan; 6 Global Health Institute, University of Antwerp, Antwerp, Belgium; 7 Policy, Planning, Budgeting and Research, Ministry of Health, Juba, Republic of South Sudan; 8 Global Health Institute, University of Antwerp, Kinsbergen Centrum, Doornstraat 331, 2610 Antwerp, Belgium

**Keywords:** Onchocerciasis, Epilepsy, Nodding syndrome, South Sudan, Ivermectin, Community directed treatment, Mundri, Maridi

## Abstract

**Background:**

Nodding syndrome (NS) is a devastating epileptic illness of unknown aetiology mainly affecting children 5–15 years of age. Head nodding distinguishes NS from other forms of epilepsy. Other manifestations of the illness include mental and physical growth retardation. Many children die as a result of falling in fires or drowning. Recently, it was shown that NS is only one of the phenotypic presentations of onchocerciasis associated epilepsy (OAE). Despite the strong epidemiological association between epilepsy and onchocerciasis, the causal mechanism is unknown. After implementation of bi-annual community directed treatment with ivermectin (CDTi) and larviciding of rivers in northern Uganda, new cases of NS have ceased, while new cases continue to emerge in South Sudanese onchocerciasis-endemic areas with an interrupted CDTi programme. This study is designed to evaluate the potential effects of bi-annual CDTi on reducing the incidence of NS/OAE in onchocerciasis-endemic areas in South Sudan.

**Methods:**

A pre-intervention door-to-door population-based household survey will be conducted in selected onchocerciasis-endemic villages in Mundri and Maridi Counties, which have a high prevalence of epilepsy. Using a validated questionnaire, the entire village will be screened by community research assistants for suspected epilepsy cases. Suspected cases will be interviewed and examined by a trained clinical officer or medical doctor who will confirm or reject the diagnosis of epilepsy. Bi-annual CDTi will be implemented in the villages and a surveillance system for epilepsy set up. By implementing an epilepsy onchocerciasis awareness campaign we expect to obtain > 90% CDTi coverage of eligible individuals. The door-to-door survey will be repeated two years after the baseline survey. The incidence of NS/OAE will be compared before and after bi-annual CDTi.

**Discussion:**

Our study is the first population-based study to evaluate the effect of bi-annual CDTi to reduce the incidence of NS/OAE. If the study demonstrates such a reduction, these findings are expected to motivate communities in onchocerciasis-endemic regions to participate in CDTi, and will encourage policy makers, funders and other stakeholders to increase their efforts to eliminate onchocerciasis.

**Electronic supplementary material:**

The online version of this article (10.1186/s40249-018-0496-2) contains supplementary material, which is available to authorized users.

## Multilingual abstract

Please see Additional file 1 for translations of the abstract into the five official working languages of the United Nations.

## Background

Nodding syndrome (NS) is a debilitating neurological condition, characterized by multiple seizure types with head nodding being predominant [1]. Head nodding manifests as spontaneous head drops towards the chest because of atonic seizures. Advanced cases of NS usually present with mental and physical growth retardation, along with other neurological abnormalities including psychiatric manifestations [2, 3]. The disease mainly develops in children 5–15 years of age [1, 4].

Initial cases of a condition consistent with NS were reported in Tanzania in the early 1960s [5], followed by South Sudan in the 1990s and 2001 [6, 7] and in northern Uganda in 2007 [4].

Several studies suggested an association of NS and other forms of epilepsy with onchocerciasis [1, 4, 8, 9]. Recently it was shown that NS is only one of the phenotypic presentations of onchocerciasis-associated epilepsy (OAE), which may include absences, atonic and myoclonic neck seizures, and Nakalanga features (growth retardation) [10]. It remains, however, unclear how onchocerciasis might cause epilepsy because so far the parasite has never been observed in the brain [9]. An auto-immune phenomenon triggered by the *Onchocerca volvulus* has been proposed as a possible explanation [11].

### Burden of NS/OAE in South Sudan

Since the first cases of NS were reported from the Western Equatoria region of South Sudan in the 1990s, the region has continued to have a high rate of new NS cases and other forms of epilepsy. In 2001, the World Health Organization (WHO) estimated the prevalence of NS to be at 4.6% among a small population in Western Equatoria, which is considered to have the highest burden of the disease [4]. During a small household survey conducted in 2013 in the same area (Mvolo), one in six children had epilepsy (NS and other forms of epilepsy), one in two households had at least one child with epilepsy, and one in three households had at least two children with epilepsy [12]. The South Sudan Relief and Rehabilitation Commission reported an epilepsy prevalence of 8.4% (including NS and other forms of epilepsy), consistent with the 2013 household survey [13].

As part of an initiation visit for a study on NS in Lui, Mundri East (June 2016), we conducted house-to-house interviews in communities living around Lui hospital. We randomly selected households and detected cases of NS and other forms of epilepsy in about half the households, with some households having more than one person with epilepsy, as was observed in Mvolo.

### Onchocerciasis in South Sudan

**Fig. 1:**
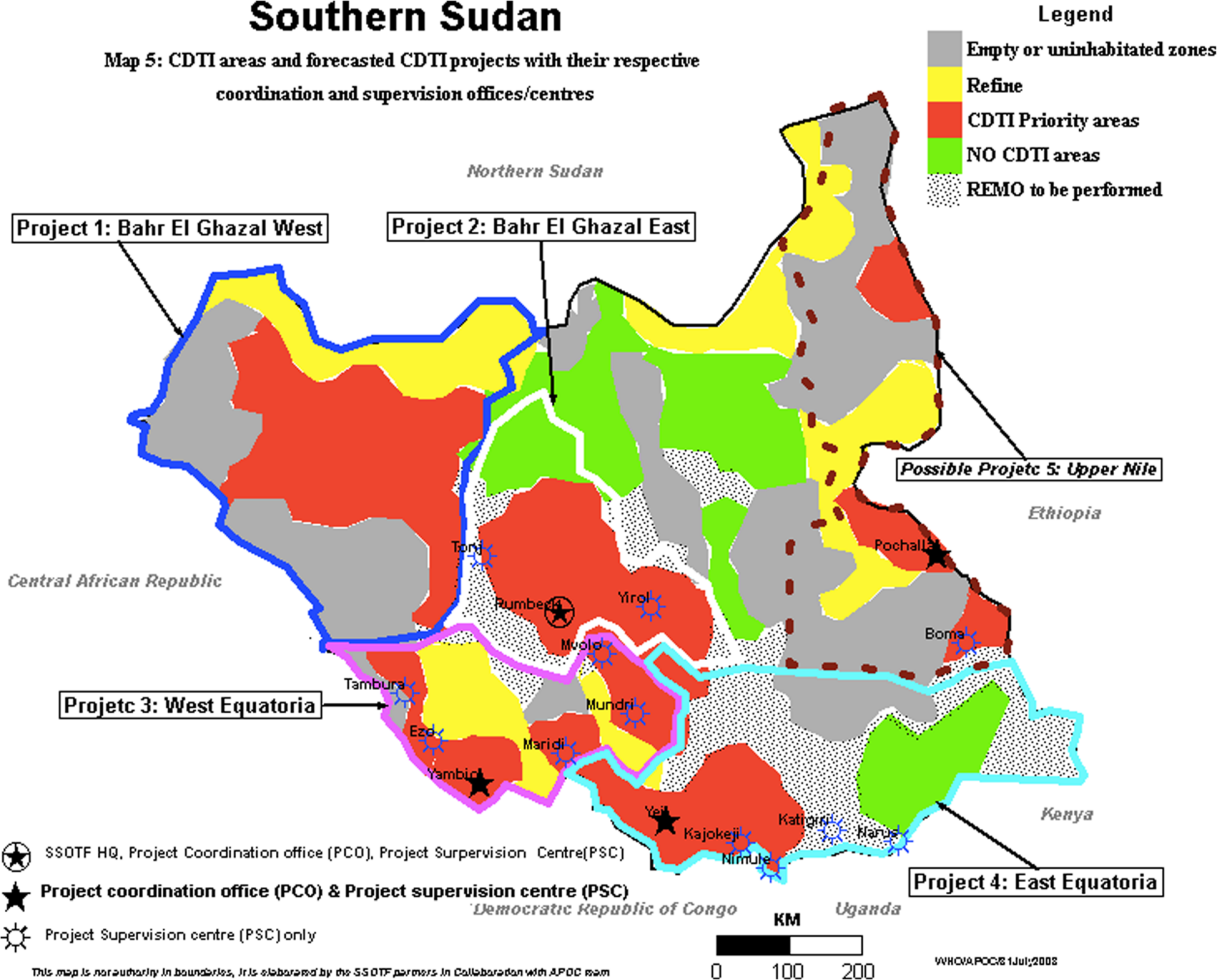
South Sudan community directed treatment with ivermectin (CDTi) areas and distribution of onchocerciasis [15]

South Sudan is amongst the highly endemic countries for onchocerciasis in Africa, with the disease being endemic in around half (49%) of the country [14]. The most highly endemic foci of onchocerciasis in South Sudan are in Western Equatoria, and the Northern and Western Bahr el Ghazal areas (Fig. 1).

In 2008, more than 80% of individuals in some villages in Western Equatoria, and the Northern and Western Bahr el Ghazal areas had palpable onchocerciasis nodules and the prevalence of river blindness was more than 12% [15]. In 2006, around 40% (4.1 million) of South Sudan’s population were at risk of onchocerciasis, of which 3.6 million were eligible for CDTi [15]. However, only 26% of the eligible population received treatment with ivermectin in the five CDTi project areas [15] (Table 1).

**Table 1:** South Sudan population treated in 2006 by community directed treatment with ivermectin (CDTi) project areas [15]

CDTi project areas	Total population eligible for CDTi	Population treated (2006)	% Treated (2006) from annual CDTi target
Western Equatoria	425 752	247 653	58
Eastern Equatoria	1 508 733	151 475	10
Eastern Bahr el Ghazal	778 920	412 021	53
Western Bahr el Ghazal	505 933	70 460	14
Upper Nile	405 994	54 766	13
Total	3 625 332	936 375	26

In 2016, WHO estimated that about 7.5 million people in South Sudan required ivermectin prophylaxis [37]

In 1950, the entomologist DJ Lewis described the distribution of onchocerciasis and its vector *Simulium damnosum* (blackfly) in Western Equatoria and highlighted the high prevalence of onchocerciasis carrying blackflies in Mvolo County [16]. These findings are consistent with recent data from the South Sudan Ministry of Health Situational Analysis on Neglected Tropical Diseases and their control, and also with reports from the African Programme for Onchocerciasis Control (APOC) [14, 15]. Because onchocerciasis in the Western Equatoria region of South Sudan has not been controlled, the associated complications of onchocerciasis (NS and other forms of epilepsy) continue to be highly endemic in these areas.

Due to scarce funding, in 1972 Sudan stopped the use of insecticides to control the onchocerciasis vector in its southern region (now South Sudan) [13]. In 2006, as the Mundri area was considered co-endemic for loiasis (caused by the nematode worm *Loa loa* and spread by its vector, the deer and mango flies), ivermectin distribution was delayed by the South Sudan Onchocerciasis Task Force [15]. However, following training of ivermectin distributors on detection and management of severe adverse events, distribution was cautiously implemented in these areas [15]. Based on results of a large-scale survey conducted between 2002 and 2010, using the rapid assessment procedure for loiasis (RAPLOA), the areas of Mundri and Maridi were not marked for loiasis risk [17]. It is possible that deforestation in the Mundri and Maridi areas has led to the disappearance of loiasis.

### Ivermectin may reduce the incidence of NS/OAE

In the NS-affected areas of South Sudan (Western Equatoria) and northern Uganda (Kitgum, Pader and Lamwo), CDTi coverage was respectively low or not implemented at all in the past because of civil conflict. However, in northern Uganda, bi-annual mass distribution of ivermectin and larviciding of rivers infested by blackflies was introduced in 2012, and subsequently no new cases of NS have been reported since 2013 [18]. In contrast, in South Sudan where insecurity as a result of civil conflict continued to prevent the implementation of CDTi, new cases of NS/OAE continue to emerge [12].

Less ivermectin use before the onset of epilepsy was reported in persons with epilepsy compared to age matched controls during the same period in Democratic Republic of the Congo [19, 20]. Since CDTi, because few new children develop OAE, an age shift of NS/epilepsy cases towards older ages (20 years or above) has been observed [21, 22]. Moreover there is anecdotal evidence that ivermectin may reduce the frequency and severity of seizures [23].

Therefore we will carry out a population-based intervention to investigate if a CDTi programme is indeed able to reduce the incidence of NS/OAE in onchocerciasis-endemic areas in South Sudan. We hypothesise that in the Mundri and Maridi Counties of South Sudan, the implementation of bi-annual CDTi with a 100% geographical coverage and a minimum of 80% coverage of ivermectin eligible individuals will reduce the incidence of NS/OAE by at least half (50%). This assumption is based on findings from Uganda where no new cases of NS appeared after bi-annual administration of ivermectin and larviciding of rivers. In South Sudan larviciding of rivers will not be done, so the effect of ivermectin alone may not be as efficient. Moreover, other forms of epilepsy not related to onchocerciasis will not be preventable by ivermectin treatment.

## Methods and analysis

### Study setting

**Fig. 2:**
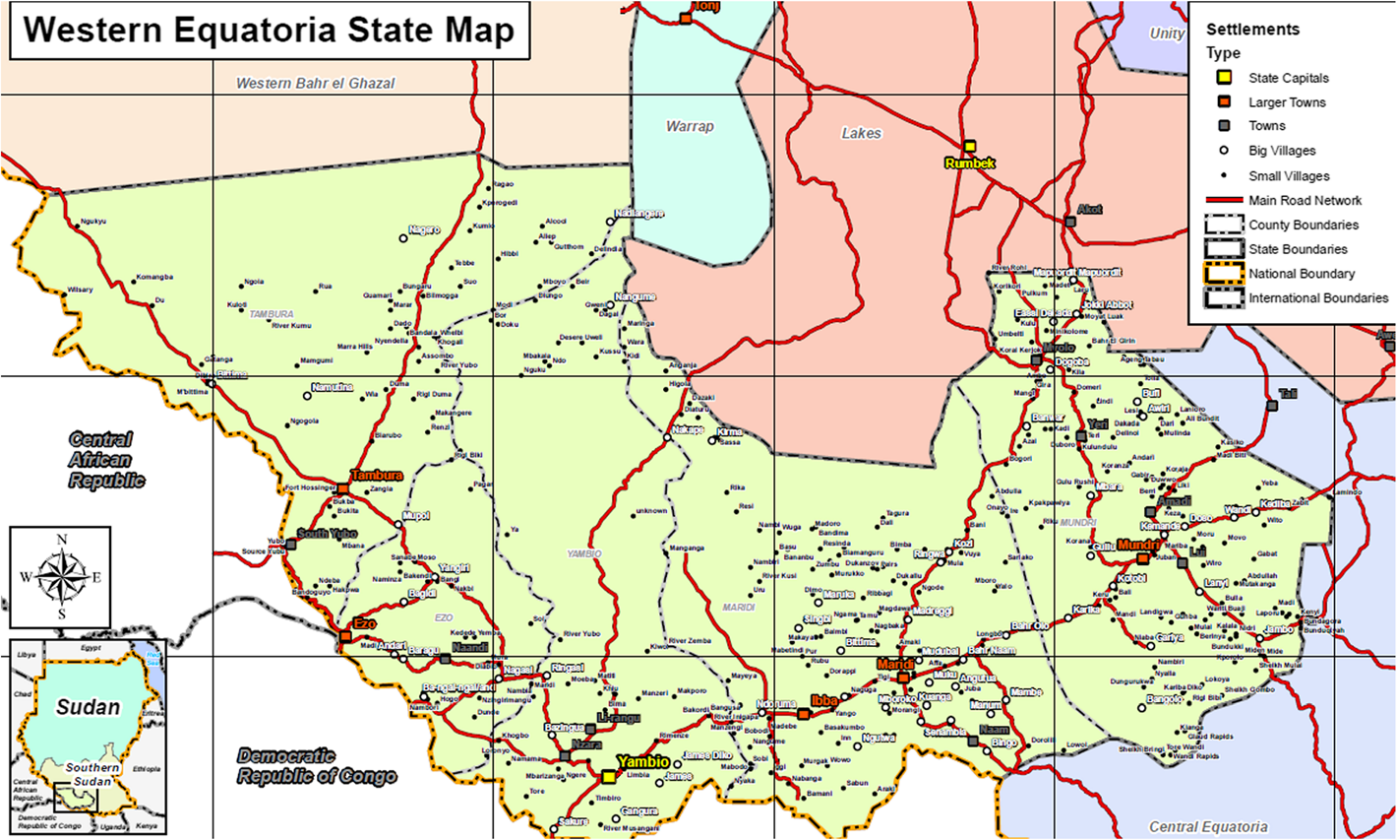
Western Equatoria State Map showing the location of Mundri and Maridi

The study will be conducted in selected villages (where the population have not been displaced as a result of civil conflict) in Mundri and Maridi Counties in the Western Equatoria region of South Sudan (Fig. 2).

Mundri and Maridi have a high prevalence of NS and other forms of epilepsy [4, 12, 13]. The population of Mundri (East and West) and Maridi Counties are estimated at 82293 and 101 065 respectively [24]; farming is the main economic and livelihood activity. A small proportion of the population practise cattle herding and fishing. Water is mainly accessed from the Yei and Maridi Rivers, which are rapidly flowing and provide sufficient breeding sites for the *Simulium* vector (blackfly) that transmits *Onchocerca volvulus*. As a result of the recent civil conflicts in South Sudan, which also affected the study area, population movement in and out of the area has been high. However, most of the population did not leave the area but rather sought shelter in nearby villages that were considered safe [25]. This might also have been the case throughout the long period of the civil war that lasted for over 20 years before the peace agreement in 2005. The study will only include participants in villages that have lived in the area for at least 5 years.

## Study design

### Prevalence and incidence of NS and other forms of epilepsy

#### Population-based survey

In order to determine the prevalence and incidence of NS and other forms of epilepsy, a population-based household survey will be conducted prior to bi-annual CDTi.

The survey will start by short questionnaire-based interviews with village leaders and village health workers to capture village demographic information.

All individuals in the selected villages will be included in a door-to-door survey. A two-step epilepsy survey will be used to identify cases of NS and other forms of epilepsy. For screening, a pre-tested validated questionnaire for epilepsy consisting of five specific questions will be used [26].

The surveys will be planned when there are no major farming activities ongoing to obtain information on all people living in the village. If at the time of the home visit nobody is at home this household will be revisited later, at least once.

##### Training of interviewers

Research assistants living in the study area will be trained on how to conduct the screening process. Questionnaires will be translated into the local language and back translated into English to ensure no loss of meaning, and pilot tested.

##### Confirmation of cases

All suspected cases will be referred to a clinical officer trained to diagnose epilepsy or a medical doctor who will visit the suspected case at home and take a detailed medical history and perform a neurological examination. The clinical officer/medical doctor will confirm or reject the diagnosis of epilepsy.

##### Definitions

A *case of epilepsy* will be defined based on the International League Against Epilepsy (ILAE) as an individual with at least two unprovoked seizures with a minimum of 24 h separating the two episodes [27].

*Active epilepsy* will be defined as a person who is taking anti-epileptic treatment or who is not on anti-epileptic treatment but had at least one episode of seizures during the last 5 years.

A *new case of epilepsy* will be defined as a person who developed the first seizures within the last 24 months preceding the baseline survey (pre-CDTi incidence), or a person who developed the first seizures after the first round of CDTi and the second study visit to the household (post-CDTi incidence).

A *suspected NS case* will be defined as a person who presented with episodes of reduced consciousness with the head dropping forward repeatedly.

*OAE* will be defined as a person meeting the following six criteria: 1) Three years of living in an onchocerciasis-endemic region; 2) High prevalence of epilepsy in the village and families with more than one child with epilepsy; 3) History of at least two unprovoked epileptic seizures at least 24 h apart; 4) No obvious cause for the epilepsy; 5) Onset of epilepsy between the ages of three and 18 years; 6) Normal psychomotor development before the onset of epilepsy.

Epilepsy caused by perinatal trauma, head trauma, meningitis/encephalitis, and cerebral malaria can be suspected through medical history taking. Epilepsy caused by cysticercosis is difficult to differentiate from OAE but in the Maridi and Mundri areas there are no pigs, therefore neurocysticercosis does not exist in these areas. It is expected that the prevalence of epilepsy not caused by onchocerciasis will be < 1.4% (the median prevalence of epilepsy in Sub-Saharan Africa) [28].

#### Follow up of epilepsy cases

The person with epilepsy will be asked to come to the health centre to initiate anti-epileptic treatment. At the health centre, the person or caretaker will be counselled about epilepsy, and anti-epileptic treatment according to WHO epilepsy guidelines and the NS management guidelines proposed by R Idro [29] will be started.

#### Community-directed treatment with ivermectin (CDTi)

Following the initial household survey, CDTi will be implemented. The CDTi will be applied twice a year (6 months apart). The standard ivermectin dose of 150 μg/kg will be used [30]. Prior to implementation of the CDTi, the study team including community workers will meet with village and community leaders to explain the concept of CDTi. Community-directed distributers (CDDs) for the ivermectin will be trained to: (i) conduct a census; (ii) use measuring sticks to establish the number of ivermectin tablets to be given; (iii) detect and treat minor ivermectin side effects; (iv) refer cases of severe adverse events to the nearest health facility; (v) fill household treatment forms; (vi) keep an ivermectin inventory; and (vii) maintain CDTi distribution records and submit a report to the study coordinator. The Onchocerciasis Elimination Programme under the department of Preventive Chemotherapy, Neglected Tropical Diseases (PC-NTDs) in the Ministry of Health will coordinate the process. Ivermectin for the study will be supplied by the department of PC NTDs. Obtaining optimal CDTi coverage will be essential in order to be able to document an effect of the intervention. Therefore we plan to implement an epilepsy onchocerciasis awareness campaign in order to obtain > 90% CDTi coverage of eligible individuals. To reach this target we will get support from the state administration, and community involvement using the state radio, churches, mosques, community meetings and using the same research assistants to make house to house visits.

### Study inclusion criteria

All members of families who have lived for at least three years in the villages and are not planning to leave the village in the next two years.

### Exclusion criteria for ivermectin treatment

Children < five years old, pregnant women, women who have been lactating less than seven days, or very ill patients are ineligible.

### Sample size

We expect a crude incidence of new onset cases of NS/OAE in the Mundri/Maridi areas to be about 1.67% per year in all children aged five to 15 years [31]. With > 90% coverage of eligible individuals and 100% geographical coverage in the study area, we expect to decrease the incidence of NS/OAE in the five to 15-year-old population by 50%. Based on the reference proportion of 0.0150 (1.5% per year), alpha 5%, statistical power 80%, 2-sided testing, a total of 3475 children aged five to 15 years old need to be included in the study.

An adaptive study design will be used to ensure an adequately powered study. The NS/OAE incidence data will be cleaned after around 3000 children have been enrolled. The required sample size will be re-calculated using the actual observed incidence rate to ensure the study is adequately powered.

#### Epilepsy surveillance

A surveillance system for detecting new cases of epilepsy in the selected villages will be set up. The research assistants who carried out the baseline survey together with community-key informants will report suspected new epilepsy cases to a nurse supervisor, who in turn will verify the case history before referring to the study clinical officer/medical doctor for confirmation and initiation of treatment.

### Research study outcomes

The prevalence of NS and other forms of epilepsy in children and adults in selected villages in Mundri and Maridi counties in 2018.

Incidence of NS/OAE after implementation of bi-annual CDTi in these villages.

### Study procedures

Pre- and post-intervention household surveys will be performed using exactly the same methodology. The pre-intervention survey will be done within two months before the start of the bi-annual CDTi. A post-intervention survey will be done 2 years later.

### Data collection, management

Data will be collected using questionnaires deployed on Open Data Kit (ODK, https://opendatakit.org/) software installed on tablet computers, which will automatically be stored on a managed Google engine server. Information will be transferred to a central database server by a trained technical data coordinator on a daily basis for quality assurance. All the study interviewers will be trained on the ODK survey system.

### Data analysis

The unit of analysis will be children between the ages of 5 to 15 years, who are most vulnerable for the development of NS/OAE. The incidence rate of NS/OAE at baseline will be calculated using incident cases in children 5 to 15 years of age during the preceding 24 months. This rate will be compared with the incidence rate of NS/OAE in the 24-month interval between the first and the second household surveys.

Data will also be collected on recent deaths that might be attributed to recent onset NS and other forms of epilepsy (household heads and community informants will be questioned). Deaths suspected to be because of NS/OAE will be considered as incident cases.

Descriptive statistics will be employed to present retention rates. Means and standard deviations will be presented for continuous outcome measures and frequencies and percentages will be presented for categorical variables. Chi-squared tests will be used to compare any group differences for categorical variables and *t*-tests will be used for continuous variables.

The incidence of NS/OAE pre- and post-intervention will be compared among those who received ivermectin twice a year over 2 years and those who missed at least one dose of ivermectin.

Conflict induced displacement of people during the study period is possible. In case of in or out migration only the data will be analysed of families who stayed for at least 5 years in the area.

## Discussion

A major strength of design of our study is that it will be the first population-based study to formally evaluate the effect of bi-annual CDTi to reduce the incidence of NS/OAE. If the study indeed demonstrates such a reduction as a result of the bi-annual CDTi, then communities in onchocerciasis-endemic regions will be motivated to take ivermectin and the results will also help convince policy makers, funders and other stakeholders to strengthen CDTi programmes.

### Limitation

A challenge for the study will be the insecurity in the region and the migration of people as a consequence. Ideally a clustered randomised controlled trial would be preferred in which one population receives ivermectin treatment and the other not. However, given the expected benefits of CDTi, withholding treatment is ethically not acceptable. Indeed in onchocerciasis-endemic regions all eligible individuals should receive ivermectin at least annually [32]. Comparing annual with bi-annual ivermectin treatment would be a possibility but this would require a very large sample size because of the relatively low incidence of epilepsy. Moreover, to achieve the elimination goal for onchocerciasis and based on success stories from the Americas and other African countries, a bi-annual CDTi strategy is preferred, especially where the onchocerciasis situation is insufficiently controlled [33–35]. Given the dramatic decrease of incidence of NS/OAE as a consequence of bi-annual CDTi and larviciding of rivers in northern Uganda [36], we expect that with an optimal bi-annual CDTi programme with > 90 coverage of eligible individuals, we will be able to show a similar decrease of incidence of NS/OAE in South Sudan.

## Additional file

Additional file 1; Multilingual abstracts in the five official working languages of the United Nations. (PDF 877 kb)

## Abbreviations

APOC: African Programme for Onchocerciasis Control
CDTi: Community directed treatment with ivermectin
IgG: Immunoglobulin G
ILAE: International League Against Epilepsy
NS: Nodding syndrome
OAE: Onchocerciasis associated epilepsy
ODK: Open Data Kit
OV: *Onchocerca volvulus*
RAPLOA: Rapid Assessment Procedure for Loiasis
WHO: World Health Organization
