# Supplementary material for: The effect of bi-annual community-directed treatment with ivermectin on the incidence of epilepsy in onchocerciasis endemic villages in South Sudan: a study protocol
Source: Infect Dis Poverty. 2018 Nov 14;7:112. doi: 10.1186/s40249-018-0496-2 (PMC6234538; doi:10.1186/s40249-018-0496-2)
Supplement: Supplementary file 1 — Multilingual abstracts in the five official working languages of the United Nations. (PDF 877 kb) [file 40249_2018_496_MOESM1_ESM.pdf]

Translation of the abstract into the five official working languages of the United Nations

تأثير العلاج النصف سنوي بالايفرمكتين الموجه للمجتمع في حالات بالصرع في القرى الموبوءة بداء كلابية الذنب في جنوب السودان: بروتوكول دراسة

جاسم عبد الفراج، ماكوي يبي لوروجا، موريش اجوك، جاكسون سونجوك، سونيا مينون، فيرنارد فيت، ريتشارد لاكو، روبرت كولبندرس

نبذة مختصرة

المعلومات الأساسية: متلازمة الايماء هو مرض صرع مدمر غير معروف السبب ويصيب الأطفال غالبا بعمر 5-15 عام. ويميز الايماء بالرأس متلازمة الايماء عن باقي أشكال الصرع. وتشمل الأعراض الأخرى للمرض تأخر النمو العقلي والجسدي. ويموت العديد من الأطفال نتيجة للتعرض للحرائق أو الغرق. وفي الآونة الأخيرة، تبين أن متلازمة الإيماء هي عبارة عن نمط من الصرع المرتبط بكلابية الذنب. وعلي الرغم من الارتباط البيولوجي بين الصرع وداء كلابية الذنب، الا أن السبب غير غير معلوم. بعد تطبيق العلاج النصف سنوي بالايفرمكتين الموجه للمجتمع ومعالجة الأنهار في شمال اوغندا، توقف ظهور حالات جديدة من متلازمة الايماء، بينما استمرت حالات جديدة في الظهور في جنوب السودان في المناطق الموبوءة بداء كلابية الذنب حيث برنامج العلاج المتقطع. وتم تصميم هذه الدراسة لتقييم الاثار المحتملة لبرنامج العلاج النصف سنوي بالايفركتين الموجه للمجتمع في تقليل معدل الاصابة بمتلازمة الايماء / الصرع المرتبط بكلابية الذنب في الموبوءة بداء كلابية الذنب في جنوب السودان.

الطرق. وسيتم اجراء مسح أسري علي السكان من عبر البيوت في القري المتوطن بها داء كلابية الذنب في مقاطعتي مندري ومريدي، حيث توجد ينتشر بها الصرع. وسيقوم مساعدي البحوث بمسح القرية باكملها باستخدام استبيان موثوق للبحث عن حالات الصرع المحتملة. وسيقوم موظف مدرب أو طبيب بمقابلة الحالات المشتبه بهم والذي سيؤكد أو ينفي تشخيص الصرع. وسيتم وضع نظام لتطبيق العلاج النصف سنوي بالايفرمكتين الموجه للمجتمع لحالات الصرع ورصد تنفيذه في القري. ونتوقع تغطية أكثر من 90 بالمائة بالعلاج النصف سنوي بالايفرمكتين للأفراد المقصودين عن طريق تنفيذ حملة التوعية لداء كلابية الذنب. وسيتم تكرار الدراسة الاستقصائية عبر المنازل بعد عامين من المسح الاولي. وسيتم مقارنة حالات بمتلازمة الايماء / الصرع المرتبط بكلابية الذنب قبل وبعد العلاج النصف سنوي بالايفرمكتين الموجه للمجتمع.

المناقشة دراستنا هي أول دراسة مبنية علي السكان لتقييم تأثير العلاج النصف سنوي بالايفرمكتين لتقليل حالات الاصابة بمتلازمة الايماء / الصرع المرتبط بكلابية الذنب. وفي حال أوضحت الدراسة تقليل معدل الحالات، من المتوقع ان تحفز هذه النتائج المجتمعات في المناطق الموبوءة بداء كلابية الذنب للمشاركة في برنامج العلاج النصف سنوي بالايفرمكتين الموجه للمجتمع وسيتم تشجيع واضعي السياسات والممولين وأصحاب المصلحة الآخرين على زيادة جهودها للقضاء على داء كلابية الذنب.

Translated from English version into Arabic by Vasser Atlem, proofread by Heba Kandel, through

TRANSLATORS WITHOUT BORDERS

伊维菌素一年两次社区指导治疗对南苏丹盘尾丝虫病流行村庄癫痫发病率的影响：研究方案

Gasim Abd-Elfarag, Makoy Yibi Logora, Morrish Ojok, Jackson Songok, Sonia Menon, Ferdinand Wit, Richard Lako, Robert Colebunders

摘要

引言：点头综合征（NS）是一种以 5–15 岁儿童为主要发病人群的、灾难性的、病因不明的癫痫病。点头与其他形式的癫痫病有显著区别。该病的其他特征表现包括智力和身体发育迟缓。患该病的许多儿童死于火灾或溺水。最近，研究表明 NS 是盘尾丝虫病相关癫痫（OAE）的表型表现之一。尽管癫痫和盘尾丝虫病之间有很强的流行病学联系，但其病因尚不清楚。在乌干达北部在河流中使用幼虫杀虫剂，并实施伊维菌素（CDTi）一年

两次的社区指导治疗后，未发生新的 NS 病例。而在南苏丹的盘尾丝虫病流行地区，因 CDTi 计划受阻，新的病例陆续出现。本研究旨在评估一年两次的 CDTi 对降低南苏丹盘尾丝虫病流行地区 NS/OAE 发病率的潜在影响。
方法：选择孟德里县和马里迪县的一些盘尾丝虫病流行且癫痫发病率很高的村庄进行干预前逐户的家庭人口调查。使用经验证的问卷进行调查，由社区研究助理对整个村庄疑似癫痫病例进行筛查。可疑病例由经过培训的临床人员或医生进行面谈和检查，对癫痫进行确诊和排除。在上述选定的村庄实施一年两次 CDTi 指导治疗，并建立癫痫监测系统。通过开展癫痫盘尾丝虫病的健康教育，我们期望符合条件人群的 CDTi 覆盖率能超过 90%。基线调查两年后将再次进行逐户调查，比较实施一年两次 CDTi 前后 NS/OAE 的发病率。
讨论：本研究是首次评估实施 CDTi 一年两次的社区指导治疗对人群 NS/OAE 发病率影响的研究。如研究表明可降低发病率，预计此发现将促使盘尾丝虫病流行区的社区参与 CDTi 一年两次的社区指导治疗，并鼓励决策者、资助者和其他利益攸关方参与消除盘尾丝虫病项目。

Translated from English version into Chinese by Xin-Yu Feng, edited by Pin Yang

CDC CHINESE CENTER FOR DISEASE CONTROL AND PREVENTION
NATIONAL INSTITUTE OF PARASITIC DISEASES

Le résultat du traitement bi-annuel par l'ivermectine sous directives communautaires (TIDC) sur l'incidence de l'épilepsie dans les villages endémiques à l'onchocercose dans la partie sud du Soudan : Un protocole d'étude

Gasim Abd-Elfarag, Makoy Yibi Logora, Morrish Ojok, Jackson Songok, Sonia Menon, Ferdinand Wit, Richard Lako, Robert Colebunders

Résumé

Origine: La maladie du hochement de tête ou maladie du tremblement (ou syndrome de Nodding) est une affection neurologique dévastatrice (forme particulière de l'épilepsie) qui affecte principalement les enfants âgés de 5 à 15 ans. Le hochement de tête différencie le syndrome de Nodding (NS) de toutes les autres formes d'épilepsie. Le syndrome de Nodding peut donner lieu à des retards de croissance et à des troubles mentaux. De nombreux enfants meurent lors de chutes pendant les incendies ou par noyade. Il a été démontré récemment que le syndrome de Nodding (NS) est seulement l'une des formes phénotypiques de l'épilepsie associée à l'onchocercose (EAO). En dépit du lien épidémiologique très important entre l'épilepsie et l'onchocercose, la cause de la maladie demeure inconnue. Après la mise en place du traitement bi-annuel à l'ivermectine sous directives communautaires (TIDC) et de l'épandage de larvicides en rivières dans la partie nord de l'Ouganda, aucun nouveau cas du syndrome de Nodding n'a été signalé dans cette partie du pays, tandis de nouveaux cas continuent d'apparaître dans les régions endémiques à l'onchocercose dans la partie sud du Soudan avec l'interruption du programme TIDC. Cette étude a pour objet d'évaluer les effets du traitement bi-annuel par l'ivermectine sous directives communautaires (TIDC) dans la réduction de l'incidence du syndrome de Nodding (NS)/épilepsie associée à l'onchocercose (EAO) dans les villages endémiques à l'onchocercose dans la partie sud du Soudan.

Méthodes: Une opération porte-à-porte visant à effectuer une enquête auprès des ménages sera conduite dans les villages endémiques à l'onchocercose dans les comtés de Mundri et de Maridi, qui révèlent une forte prévalence de l'épilepsie. Aux fins de diagnostiquer des cas d'épilepsie, des assistants de recherche dans la communauté contrôleront l'ensemble de la population des villages au moyen d'un questionnaire validé. Les personnes suspectées d'être atteintes de la maladie seront interrogées et examinées par un agent clinicien qualifié ou par un médecin, qui confirmera ou réfutera le diagnostic de l'épilepsie. Le traitement bi-annuel par l'ivermectine sous directives communautaires (TIDC) ainsi qu'un

système de surveillance pour les formes particulières d'épilepsie seront mis en place. En mettant en oeuvre une campagne de sensibilisation de l'épilepsie associée à l'onchocercose, nous entendons obtenir plus de 90% de couverture santé du traitement bi-annuel par l'ivermectine sous directives communautaires (TIDC) pour les personnes admissibles. L'enquête porte-à-porte sera renouvelée deux ans après l'enquête initiale. La prévalence du syndrome de Nodding(NS)/épilepsie associée à l'onchocercose (EAO) sera comparée avant et après le traitement bi-annuel par l'ivermectine sous directives communautaires (TIDC).

Discussion: Notre étude constitue la première enquête effectuée auprès de la population aux fins d'évaluer les résultats du traitement bi- annuel par l'ivermectine sous directives communautaires (TIDC) pour réduire la prévalence de syndrome de Nodding (NS)/épilepsie associée à l'onchocercose (EAO). Si notre étude révèle une diminution des cas de la maladie, nous espérons que ces découvertes motiveront les communautés vivant dans les zones endémiques à l'onchocercose de participer au traitement bi-annuel par l'ivermectine sous directives communautaires (TIDC) et encourageront les décideurs, les bailleurs et les autres parties prenantes à accroître leurs efforts dans l'éradication de l'onchocercose.

Translated from English version into French by Vero Marie, proofread by Chrystelle C. Ngateu, through

TRANSLATORS
WITHOUT BORDERS

Эффект от проводимого два раза в год на местном уровне лечения ивермектином на заболеваемость эпилепсией в эндемичных деревнях Южного Судана по онхоцеркозу: протокол исследования

Гасим Абд-Элфараг, Макой Иби Логора, Морриш Ожок, Джексон Сонгок, Соня Менон, Фердинанд Вит, Ричард Лако, Роберт Коленбандерс

Аннотация

Исходная информация: Кивательный синдром (КС) представляет собой разрушительное по своему действию эпилептическое заболевание неизвестной этиологии, в основном поражающее детей в возрасте от 5 до 15 лет. КС отличается от других форм эпилепсии характерным симптомом кивания головой. К другим проявлениям болезни относится задержка психического и физического развития. Многие дети погибают в результате падения в костры или утопления. Недавно было обнаружено, что КС — это лишь одно из фенотипических проявлений эпилепсии, ассоциированной с онхоцеркозом (ЭАО). Несмотря на сильную эпидемиологическую связь между эпилепсией и онхоцеркозом, причинно-следственный механизм неизвестен. В результате имплементации проводимого два раза в год и осуществляемого на местном уровне лечения ивермектином (CDTi) и ларвицидной обработки рек в северной Уганде, в этом регионе прекратилось возникновение новых случаев заболевания КС, в то время как в районах Южного Судана с эндемичным уровнем онхоцеркоза, где имплементация программы CDTi была прервана, новые случаи заболевания продолжают возникать. Целью этого исследования является определение потенциального влияния проводимого два раза в год и осуществляемого на местном уровне лечения ивермектином на частоту заболеваемости КС и ЭАО в районах Южного Судана с эндемичным уровнем онхоцеркоза.

Методология: В преддверии имплементации лечения в отдельных деревнях с эндемичным уровнем онхоцеркоза в округах Мундри и Мариди, в которых отмечается высокая распространенность эпилепсии, будет проведён поквартирный опрос популяционного масштаба. С помощью утверждённого вопросника специалистами будет произведено обследование жителей деревни на предмет возможных случаев эпилепсии. Затем специально обученные сотрудники или врачи проведут беседу и осмотр лиц с подозрениями на болезнь для окончательного подтверждения или опровержения диагноза эпилепсии. Два раза в год в деревнях будет проводиться осуществляемое на местном уровне лечение ивермектином, а также будет создана система эпидемиологического надзора за эпилепсией. В результате проведения кампании по привлечению внимания общественности к проблеме ЭАО, мы рассчитываем, что более 90% лиц, имеющих на то показания, смогут получить доступ к осуществляемому на местном уровне лечению ивермектином. Сквозной опрос населения будет проведен повторно через два года после базового исследования. Будут сравниваться показатели частоты заболеваемости КС/ЭАО до и после проводимого два раза в год и осуществляемого на местном уровне лечения ивермектином.
Обсуждение: Наше исследование представляет собой первое исследование популяционного масштаба по определению влияния проводимого два раза в год и осуществляемого на местном уровне лечения ивермектином на сокращение частоты заболеваемости КС/ЭАО. Если исследование продемонстрирует сокращение частоты заболеваемости, то эти результаты послужат стимулом для лиц из регионов с эндемичным уровнем онхоцеркоза к участию в программе CDTi и будут способствовать тому, что политики, спонсоры и другие заинтересованные стороны активизируют свои усилия в борьбе против онхоцеркоза.

Translated from English version into Russian by Ekaterina Grebenshchikova, proofread by Liudmila Tomanek, through

TRANSLATORS
WITHOUT BORDERS

Efecto del tratamiento con Viacmertina bianual dirigido a la comunidad sobre la incidencia de la epilepsia en pueblos con oncocercosis endémica en Sudán del Sur: un protocolo de estudio

Gasim Abd-Elfarag, Makoy Yibi Logora, Morrish Ojok, Jackson Songok, Sonia Menon, Ferdinand Wit, Richard Lako, Robert Colebunders

Resumen
Información general: El síndrome del cabeceo (NS por sus siglas en inglés) es una enfermedad epiléptica devastadora de la cual no se conoce su etiología y que afecta mayormente a niños de entre 5 a 15 años. El cabeceo diferencia este sindrome de otros tipos de epilepsia. Otra de las manifestaciones de esta enfermedad es un retraso en el crecimiento físico y mental. Muchos niños mueren por ahogamiento o por caerse a los fuegos. Se ha comprobado recientemente que el NS es una de las presentaciones fenotípicas de la epilepsia asociada a la oncocercosis (OAE por sus siglas en inglés). A pesar de la estrecha relación epidemiológica entre la epilepsia y la oncocercosis, el mecanismo causal no se conoce. Despues de implementar el tratamiento con Viacmertina bianual dirigido a la comunidad (CDTi por sus siglas en inglés) y larvicida de los ríos en el norte de Uganda, han cesado los casos de NS, mientras que continúan emergiendo nuevos casos en Sudán del Sur en áreas de oncocercosis endémica con un programa de CDTi ininterrumpido. Este estudio

pretende evaluar los efectos potenciales del CDTi bianual en la reducción de la indicencia del NS y la OAE en áreas de oncocercosis endémica de Sudán del Sur.

Métodos: Se llevará a cabo una encuesta de preintervención puerta por puerta en determinados pueblos seleccionados de los condados Mundri y Maridi, donde la encocercosis endémica está muy extendida. Mediante un cuestionario validado, un grupo de asistentes de investigación comunitarios examinará a todo el pueblo en busca de casos sospechosos de epilepsia. Si se encuentran casos sospechosos, estos serán examinados y entrevistados por un agente con formación clínica o un médico que confirmará o rechazará el diagnóstico de epilepsia. El CDTi bianual se implementará en los pueblos y se implantará un sistema de viligancia para la epilesia. Con la implementación de una campaña de concienciación sobre la oncocercosis y la epilepsia, esperamos obtener una cobertura de CDTi de > 90% para individuos afectados. La encuesta puerta por puerta se repetirá dos años después de la encuesta base. La incidencia del NS/OAE se comparará antes y después del CDTi bianual.

Consideraciones finales: Nuestro estudio es el primer estudio poblacional que evalúa el efecto del CDTi bianual para reducir la incidencia del NS/OAE. Si el estudio llega a demostrar esa reducción, se espera que estos hallazgos sirvan para motivar a las comunidades en las regiones con oncocercosis endémica a participar en el CDTi y también animará a los actores políticos, financiadores y otros accionistas a hacer un mayor esfuerzo para erradicar la oncocercosis.

Translated from English version into Spanish by Clarines, proofread by Antonio Sanchez, through

TRANSLATORS
WITHOUT BORDERS
